# Identifying latent profiles of emotional labor and exploring their links to psychological resilience among tertiary hospital nurses

**DOI:** 10.3389/fpsyg.2025.1742147

**Published:** 2026-01-12

**Authors:** Zhi Zeng, Guiqiong Xie, Yazhi He, Sumei Zhou

**Affiliations:** 1 Department of Gastroenterology, Deyang People’s Hospital, Deyang, Sichuan, China; 2 Department of Neurosurgery, Deyang People’s Hospital, Deyang, Sichuan, China

**Keywords:** emotional labor, latent profile analysis, psychological resilience, nurses, tertiary hospitals, cross-sectional study

## Abstract

**Background:**

Nurses frequently engage in high levels of emotional labor, which, when sustained, may be detrimental to their psychological well-being. However, the way nurses regulate emotions is heterogeneous. Identifying distinct emotional labor profiles and examining their psychological associations is crucial for developing tailored interventions.

**Objective:**

This study aimed to identify latent profiles of emotional labor among nurses in tertiary hospitals and investigate their associations with psychological resilience.

**Methods:**

A cross-sectional survey was conducted from March to May 2025 among 458 registered nurses across eight tertiary hospitals in Sichuan Province, China. Data were collected using a general demographic questionnaire, the Emotional Labor Scale, and the Psychological Resilience Scale. Latent Profile Analysis (LPA) was employed to identify distinct emotional labor profiles. One-way ANOVA was used to compare psychological resilience across profiles, and a multivariate logistic regression model was constructed to explore independent predictors of emotional labor categories.

**Results:**

A total of 458 valid responses were analyzed. Three distinct emotional labor profiles were identified: Surface Acting-Suppression Type (C1, 30.3%), Deep Acting Type (C2, 45.4%), and Natural Engagement Type (C3, 24.2%). Multivariate logistic regression revealed that gender, age, employment type, monthly night shifts, salary satisfaction, and psychological resilience were significant predictors of emotional labor classification. Psychological resilience significantly differed across all profile comparisons: C1 vs. C2, C1 vs. C3, and C2 vs. C3 (*p* < 0.05).

**Conclusion:**

Emotional labor among nurses exhibits notable latent heterogeneity, with psychological resilience varying significantly across profile types. Tailored interventions are recommended based on emotional labor typologies to enhance psychological resilience and organizational support, thereby improving emotional labor management and promoting sustainable occupational health among nurses.

## 1 Introduction

With the implementation of the “Healthy China 2030” strategy, higher standards have been set for both the quality of medical services and the occupational health of nursing staff (Central Committee of the Communist Party of China and The State Council, 2016). As the largest and most patient-facing group within the healthcare system, nurses are responsible not only for intensive clinical tasks but also for sustained emotional regulation and affective expression (Gu et al., 2019; Ren et al., 2024). This high-intensity, often invisible emotional labor imposes a significant psychological burden and has become a critical risk factor affecting nurses’ mental health (Sun et al., 2025).

Against the backdrop of a deepening “patient-centered” care philosophy and the bio-psycho-social medical model, the role of nurses is shifting from mere “task executors” to “emotional caregivers.” Emotional labor is increasingly recognized as an essential psychological process in nursing, directly linked to job stability, work satisfaction, and psychological well-being. The term was first introduced by Hochschild in 1983 (Grandey and Melloy, 2017), and later conceptualized by Grandey and others into three dimensions: surface acting, deep acting, and natural expression (Grandey, 2000). Surface acting involves suppressing true emotions and displaying organization-expected expressions, which can lead to emotional exhaustion and role detachment. In contrast, deep acting aligns internal feelings with external expression and fosters professional identity. Natural expression reflects genuine and congruent emotions, and is considered the most adaptive and psychologically healthy strategy (Nguyen et al., 2022; Céleste and Brotheridge, 2002).

Previous studies have demonstrated a strong association between emotional labor strategies and nurses’ psychological well-being and professional behavior. For instance, Brotheridge and colleagues found that frequent use of surface acting significantly increased levels of emotional exhaustion and the intention to leave the profession (Céleste and Brotheridge, 2002). Similarly, research by Chen indicated that a high emotional labor load was closely related to resource depletion and occupational burnout (Chen et al., 2024). In a separate investigation, Glomb identified nursing as the fifth most emotionally demanding occupation among 15 surveyed professions, underscoring the considerable emotional regulation burden faced by nurses (Glomb et al., 2004). Despite these findings, current nursing management practices still lack adequate and targeted intervention tools grounded in empirical classification systems.

The Conservation of Resources (COR) theory offers a valuable conceptual framework for explaining the connection between emotional labor and mental health. This theory emphasizes that individuals tend to preserve and replenish key personal resources, such as emotional energy, time, and social support, especially when confronted with stressors (Prapanjaroensin et al., 2017). Surface acting, which typically does not generate emotional reciprocity, often leads to resource depletion. In contrast, deep acting and natural expression are more likely to produce positive emotional feedback, facilitate the restoration of internal resources, and alleviate emotional exhaustion (Chen et al., 2024; Feng et al., 2024).

Psychological resilience is regarded as a crucial internal asset that enables individuals to maintain stable functioning and adapt effectively when facing adversity (Yu et al., 2025). Nurses with high resilience are generally more capable of regulating their emotions and are more inclined to adopt deep acting or natural expression strategies. These individuals also tend to display stronger resource recovery abilities and a higher level of commitment to their profession (Wang et al., 2012; Foster et al., 2024). Although existing literature has acknowledged the correlation between psychological resilience and emotional labor (Wang et al., 2012), few studies have explored this relationship using person-centered approaches such as latent profile analysis (LPA). The absence of such analyses has limited a more nuanced understanding of how nurses differentially regulate emotions under similar occupational demands.

Most prior research has been based on variable-centered models, which primarily examine group-level trends and linear associations. This approach overlooks the potential heterogeneity that exists within the nursing population (Muthén and Muthén, 2000). In practice, nurses do not respond uniformly to emotionally demanding situations. Instead, their emotional regulation styles may vary considerably, suggesting the existence of unobserved subgroups. LPA, a person-centered statistical technique, can identify these hidden subpopulations based on patterns in individual behavior. This method can help reveal the diversity of emotional labor strategies and support the development of tiered management strategies and intervention programs suited to each subgroup (Howard and Hoffman, 2017; Su et al., 2025).

In this context, the present study applied LPA to identify latent profiles of emotional labor among nurses in tertiary hospitals. The analysis further examined variations in psychological resilience and demographic characteristics across these profiles. In addition, multivariate logistic regression was used to explore the independent predictors of profile membership. The findings aim to provide empirical evidence for differentiated psychological support and emotional labor management, thereby contributing to improved nurse well-being and more sustainable organizational development.

## 2 Methods

### 2.1 Study design and setting

This study employed a cross-sectional survey design and was conducted between March and May 2025 in eight tertiary hospitals located in Sichuan Province, China. Given that the target population comprised currently employed clinical nurses who often have demanding work schedules and are difficult to access for research purposes, this study adopted a convenience sampling method to facilitate participant recruitment.

### 2.2 Sampling and participants

The inclusion criteria were as follows: (1) aged 18 years or older and holding a valid nursing license; (2) having at least 1 year of clinical work experience; and (3) providing informed consent and participating voluntarily.

Exclusion criteria included: (1) nurses in internship or advanced training programs, or those primarily engaged in administrative roles; (2) individuals on long-term leave, such as medical or maternity leave; and (3) those who had recently experienced major life events.

Among the 458 clinical nurses, 59 were male (12.9%) and 399 were female (87.1%). In terms of age, 76 (16.6%) were aged ≤25 years, 123 (26.9%) were aged 26–35 years, 151 (33.0%) were aged 36–45 years, and 108 (23.6%) were aged over 45 years. Regarding marital status, 316 participants (69.0%) were married, 93 (20.3%) were unmarried, and 49 (10.7%) fell into other categories. In terms of educational background, 49 (10.7%) held an associate degree, 349 (76.2%) had a bachelor’s degree, and 60 (13.1%) had a master’s degree or higher. With respect to employment type, 330 (72.1%) were contract nurses and 128 (27.9%) were permanently employed. As for professional title, 213 (46.5%) were junior-level, 195 (42.6%) were intermediate-level, and 50 (10.9%) were senior-level. More details can be found in Table 1.

**Table 1:** Demographic characteristics of nurses and univariate analysis across emotional labor profiles [*n* = 458, *n* (%)].

Variables	Type	*N*	Surface acting-suppression type (*n* = 139)	Deep acting type (*n* = 208)	Natural engagement type (*n* = 111)	*χ^2^*/*F*	*p*
Gender	Male	59(12.9)	29(20.9)	20(9.6)	10(9.0)	11.351	0.030
	Female	399(87.1)	110(79.1)	188(90.4)	101(91.0)		
Age	≤25 years	76(16.6)	24(17.3)	32(15.4)	20(18.0)	21.194	0.002
	26-35 years	123(26.9)	25(18.0)	66(31.7)	32(28.8)		
	36-45 years	151(33.0)	44(31.7)	78(37.5)	29(26.1)		
	>45 years	108(23.6)	46(33.1)	32(15.4)	30(27.0)		
Department	Outpatient	53(11.6)	21(15.1)	22(10.6)	10(9.0)	11.677	0.766
	Internal Medicine	79(17.2)	25(18.0)	36(17.3)	18(16.2)		
	Surgery	62(13.5)	19(13.7)	29(13.9)	14(12.6)		
	Obstetrics/gynecology	56(12.2)	19(13.7)	24(11.5)	13(11.7)		
	Pediatrics/neonatology	39(8.5)	10(7.2)	17(8.2)	12(10.8)		
	Operating Room/anesthesia Unit	23(5.0)	6(4.3)	12(5.8)	5(4.5)		
	ICU	30(6.6)	7(5.0)	15(7.2)	8(7.2)		
	Emergency	91(19.9)	23(16.5)	39(18.8)	29(26.1)		
	Infectious Diseases	25(5.5)	9(6.5)	14(6.7)	2(1.8)		
Marital status	Married	316(69.0)	86(61.9)	152(73.1)	78(70.3)	11.820	0.019
	Unmarried	93(20.3)	28(20.1)	42(20.2)	23(20.7)		
	Others	49(10.7)	25(18.0)	14(6.7)	10(9.0)		
Educational attainment	Associate degree	49(10.7)	11(7.9)	29(13.9)	9(8.1)	5.441	0.245
	Bachelor’s degree	349(76.2)	112(80.6)	153(73.6)	84(75.7)		
	Master’s degree or above	60(13.1)	16(11.5)	26(12.5)	18(16.2)		
Employment Type	Contract	330(72.1)	89(64.0)	158(76.0)	83(74.8)	6.431	0.040
	Permanent	128(27.9)	50(36.0)	50(24.0)	28(25.2)		
Professional title	Junior	213(46.5)	69(49.6)	95(45.7)	49(44.1)	3.368	0.498
	Intermediate	195(42.6)	58(41.7)	85(40.9)	52(46.8)		
	Senior	50(10.9)	12(8.6)	28(13.5)	10(9.0)		
Years of clinical	<2 years	46(10.0)	14(10.1)	20(9.6)	12(10.8)	7.588	0.270
	2–5 years	42(9.2)	12(8.6)	17(8.2)	13(11.7)		
	5–10 years	150(32.8)	35(25.2)	77(37.0)	38(34.2)		
	>10 years	220(48)	78(56.1)	94(45.2)	48(43.2)		
Monthly night shifts	0–4	169(36.9)	61(43.9)	81(38.9)	27(24.3)	19.285	<0.001
	4–8	205(44.8)	60(43.2)	95(45.7)	50(45.0)		
	≥8	84(18.3)	18(12.9)	32(15.4)	34(30.6)		
Salary satisfaction	Dissatisfied	61(13.3)	10(7.2)	28(13.5)	23(20.7)	18.609	<0.001
	Neutral	223(48.7)	68(48.9)	93(44.7)	62(55.9)		
	Satisfied	174(38.0)	61(43.9)	87(41.8)	26(23.4)		

### 2.3 Ethical considerations

The study was approved by the Ethics Committee of Deyang People’s Hospital (Approval No. 2023–04-083-K01). Written informed consent was obtained from all participants prior to data collection.

### 2.4 Sample size calculation

Sample size estimation was performed using G*Power 3.1 software. The analysis was based on a multivariate logistic regression model, assuming a medium effect size of 0.25, a two-tailed significance level of *α* = 0.05, and a statistical power of 90%, with 10 predictors included. The calculated minimum required sample size was 402 participants.

The questionnaire was sent to 493 individuals. After excluding 35 responses due to evident response patterns or logical inconsistencies, a total of 458 valid responses for analysis, yielding a valid response rate of 92.9%. This sample size met the statistical requirements for the planned analyses.

### 2.5 Data collection procedures

Data were collected using a structured online questionnaire administered via the Wenjuanxing electronic platform. The Nursing Departments and head nurses at the participating hospitals coordinated the distribution of the questionnaire. The online questionnaire format was selected to accommodate the irregular schedules of clinical nurses,enhance response rates and ensure anonymity, and reduce social desirability bias. After receiving a standardized explanation and being fully informed of the study purpose, participants completed the questionnaire independently during their non-working hours.

To ensure data quality, the following measures were implemented:

(1) Mandatory response settings were applied to prevent missing data;
(2) A minimum response time of 5 min was required to exclude abnormally fast submissions;
(3) IP address and device restrictions were activated to prevent duplicate entries.

After collection, all responses were independently reviewed by two researchers. Responses exhibiting logical inconsistencies or invalid response patterns were excluded to ensure data validity.

### 2.6 Measurement instruments

(1) General Demographic Questionnaire

Participants’ demographic and job-related characteristics were collected through a structured self-report form. Variables included gender, age, department, marital status, educational background, professional title, employment type, years of clinica, monthly night shifts, and salary satisfaction.

(2) Emotional Labor Scale (ELS)

The Chinese version of the Emotional Labor Scale, revised by Luo Hong, was used to assess the emotional regulation strategies employed by nurses in their professional roles (Luo et al., 2008). The scale consists of 14 items across three dimensions: surface acting, deep acting, and natural expression. Each item is rated on a 5-point Likert scale (1 = strongly disagree, 5 = strongly agree), with all items positively scored. Higher scores indicate greater use of the respective strategy. No total score is calculated for the Emotional Labor Scale; instead, subscale scores are computed separately, with the highest subscale score indicating the most frequently used emotional labor strategy by the respondent. The ELS has demonstrated strong reliability and validity in nursing populations (Dong and Chen, 2025; Tian et al., 2025). In the present study, the overall Cronbach’s alpha for the scale was 0.900, with subscale reliabilities of 0.892 for surface acting, 0.800 for deep acting, and 0.819 for natural expression. In addition, the KMO value exceeded 0.70, and Bartlett’s test of sphericity reached statistical significance (*p* < 0.05), indicating acceptable sampling adequacy and construct validity.

(3) Connor-Davidson Resilience Scale (CD-RISC)

The Chinese version of the Connor-Davidson Resilience Scale, revised by Yu XiaoNan, was adopted to assess psychological resilience (Yu and Zhang, 2007). The scale comprises 25 items grouped into three dimensions: tenacity (13 items), strength (8 items), and optimism (4 items). Each item is rated on a 5-point scale (0 = never, 4 = always), yielding a total score ranging from 0 to 100. Higher scores reflect stronger capacity to cope with and recover from stress or adversity. In this study, the overall Cronbach’s alpha coefficient was 0.927. The reliability coefficients for the three dimensions were 0.923 for tenacity, 0.875 for strength, and 0.870 for optimism. The KMO value was above 0.80, and Bartlett’s test of sphericity was statistically significant (*p* < 0.05), further supporting the scale’s structural validity in this sample.

To assess the potential influence of common method variance (CMV) due to the use of self-report questionnaires, Harman’s single-factor test was conducted. All items from the Emotional Labor Scale and the Psychological Resilience Scale were included in an unrotated exploratory factor analysis. The results showed that the first unrotated factor accounted for 28.44% of the total variance, which is well below the commonly accepted threshold of 40%. These findings suggest that CMV is unlikely to pose a significant threat to the validity of the results in this study.

### 2.7 Statistical analysis

After confirming the accuracy of the data through double entry verification, all responses were imported into SPSS version 26.0 and Mplus version 8.3 for analysis.

(1) Latent Profile Analysis (LPA)

Latent Profile Analysis was conducted using Mplus 8.3 to identify distinct emotional labor profiles based on item-level scores from the Emotional Labor Scale. The modeling process began with a single-class model and progressively increased the number of classes. Model fit was evaluated using the Akaike Information Criterion (AIC), Bayesian Information Criterion (BIC), and sample-size adjusted BIC (aBIC), with lower values indicating better model fit. Classification accuracy was assessed using entropy, where values closer to 1 reflect higher classification precision. Model comparisons were further supported by the Lo–Mendell–Rubin Adjusted Likelihood Ratio Test (LMRT) and the Bootstrap Likelihood Ratio Test (BLRT). A statistically significant improvement in model fit was indicated by *p* < 0.05.

(2) Comparative Analyses between Emotional Labor Profiles

Based on the emotional labor profiles identified through latent profile analysis (LPA), comparisons of psychological resilience and demographic characteristics across the three groups were conducted using SPSS version 26.0. For categorical variables, the chi-square test was used. For continuous variables (e.g., age, years of clinical experience, and monthly night shifts), one-way analysis of variance (ANOVA) was applied to compare mean differences across profiles.

Prior to conducting ANOVA, the assumptions of normality and homogeneity of variance were assessed using the Shapiro–Wilk test and Levene’s test, respectively. These assumptions were sufficiently met, supporting the use of parametric analysis for group comparisons. All group comparisons were conducted based on the measurement level of the variables and the distributional properties of the data.

(3) Multivariate Analysis

A multinomial logistic regression model was constructed using emotional labor categories (identified via LPA) as the dependent variable. Independent variables were selected based on statistically significant group differences observed in the preliminary analyses. Categorical variables were entered into the model according to their measurement level. Nominal variables were dummy-coded automatically by SPSS, while ordinal variables were treated as ordered categorical variables. Multicollinearity diagnostics were performed using variance inflation factors (VIFs), and sample adequacy for multinomial logistic regression was evaluated based on standard criteria. All statistical tests were two-tailed, and *p* < 0.05 was considered statistically significant.

## 3 Results

### 3.1 Scores on the emotional labor scale and psychological resilience scale

Values are presented as mean ± standard deviation (SD), based on distributional properties. The total score on the Emotional Labor Scale among nurses was 49.57 ± 9.31, indicating a moderately high level of emotional labor. Specifically, the mean score for the surface acting dimension was 24.86 ± 5.57, for deep acting was 13.91 ± 3.20, and for natural expression was 10.81 ± 2.52.

The total score on the Psychological Resilience Scale was 63.03 ± 15.33. Among its subdimensions, the mean score for tenacity was 33.59 ± 9.40, for strength was 19.51 ± 6.11, and for optimism was 9.93 ± 3.42.

### 3.2 Latent profile analysis of emotional labor among nurses

Using the 14 item scores from the Emotional Labor Scale as observed indicators, latent profile models with one to five classes were fitted. Model fit statistics are shown in Table 2. As the number of classes increased, the values of the Akaike Information Criterion (AIC), Bayesian Information Criterion (BIC), and adjusted BIC (aBIC) decreased gradually, and the entropy values remained above 0.80 across all models.

**Table 2:** Model fit indices for latent profile models of nurses’ emotional labor.

Class	AIC	BIC	aBIC	*P*		Enproty	Number of classes	Class probability (%)
				LMRT	BLRT			
1	18297.675	18413.227	18324.364	–	–	–	–	–
2	16592.628	16770.084	16633.615	0.0000	0.0000	0.913	206/252	44.978/55.022
3	16199.475	16438.833	16254.759	0.0260	0.0000	0.863	139/208/111	30.349/45.415/24.236
4	16004.858	16306.119	16074.439	0.5091	0.0000	0.876	41/174/158/85	8.952/37.991/34.498/18.559
5	15804.857	16168.021	15888.736	0.1401	0.0000	0.876	73/28/169/103/85	15.939/6.114/36.900/22.489/18.559

**Figure 1:**
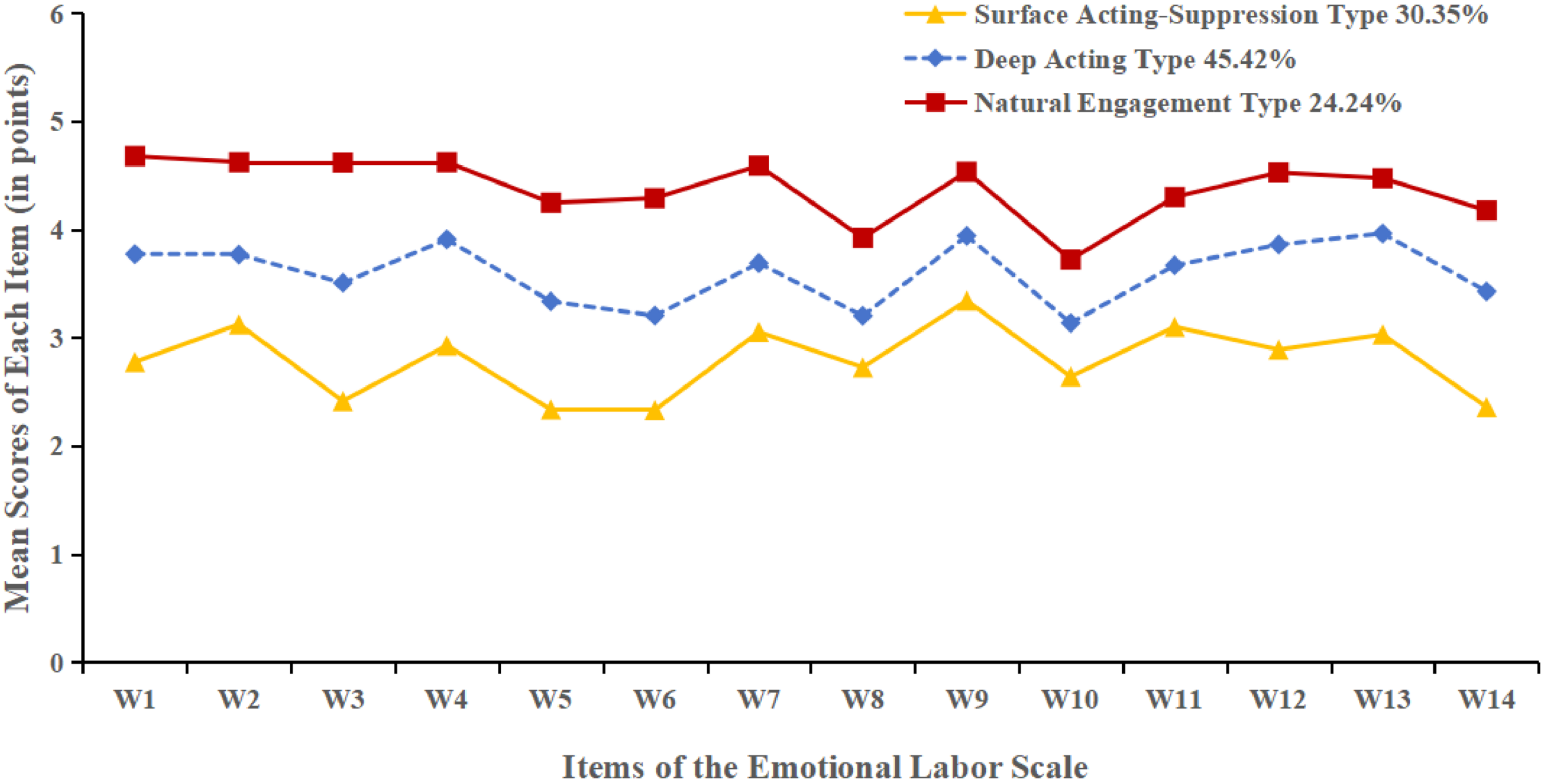
Distribution of emotional labor characteristics across the three latent profiles among nurses.

However, in the four-class model, the *p* value of the Lo–Mendell–Rubin Adjusted Likelihood Ratio Test (LMRT) was 0.509, which did not reach statistical significance, indicating poor model fit. Considering that the three-class model had a relatively high entropy value, with both LMRT and Bootstrap Likelihood Ratio Test (BLRT) reaching significance, and the average posterior probabilities for each class ranging from 91.05 to 93.24%, the classification was considered stable and clearly distinguishable. Therefore, the three-class model was selected as the optimal solution, identifying three distinct latent profiles of emotional labor (C1, C2, and C3). The mean score trends across the 14 items for each class are illustrated in Figure 1.

Class C1 had the lowest scores across all three dimensions, with a relatively high score in surface acting but the lowest scores in deep acting and natural expression. This suggests that nurses in this class primarily relied on surface-level emotional regulation, with a clear mismatch between expressed and internal emotions. Such a pattern may indicate emotional suppression or detachment and a higher risk of emotional exhaustion. This group comprised 139 nurses (30.35%) and was labeled the “Surface Acting–Suppression Type.”

Class C2 showed moderately high scores across all three emotional labor strategies. These nurses tended to actively regulate their emotions to align with job expectations, demonstrating strong emotional adaptability. This profile was considered a typical functional regulation type and included 208 nurses (45.42%). It was named the “Deep Acting Type.”

Class C3 reported the highest scores in all dimensions, particularly in natural expression. This indicates that nurses in this group were able to express their emotions authentically and consistently, reflecting high emotional congruence and strong professional engagement. A total of 111 nurses (24.24%) fell into this group, which was defined as the “Natural Engagement Type.”

### 3.3 Sociodemographic characteristics by emotional labor profile

Significant differences were observed among the three latent emotional labor profiles in terms of gender, age, marital status, employment type, monthly night shifts, and salary satisfaction (*p* < 0.05). Detailed results are presented in Table 1.

### 3.4 Comparison of psychological resilience across emotional labor profiles

Further comparisons were conducted to examine differences in psychological resilience among the three profile groups. The findings indicated that nurses in different emotional labor profiles showed statistically significant differences in total psychological resilience scores as well as in all three subdimensions: tenacity, strength, and optimism (all *p* < 0.05; see Table 3).

**Table 3:** Comparison of psychological resilience scores between latent emotional labour profiles among nurses.

Type	Score	Surface acting-suppression type	Deep acting type	Natural engagement type	*F*	*η^2^*	*P*	*Post hoc* comparison
Tenacity	33.59 ± 9.40	38.09 ± 7.11	33.03 ± 9.54	28.99 ± 9.20	33.91	0.130	<0.001	C1 > C2 > C3
Strength	19.51 ± 6.11	22.45 ± 5.21	19.14 ± 6.18	16.52 ± 5.41	33.94	0.130	<0.001	C1 > C2 > C3
Optimism	9.93 ± 3.42	10.78 ± 3.40	9.90 ± 3.46	8.94 ± 3.10	9.30	0.039	<0.001	C1 > C2 > C3
Psychological resilience	63.03 ± 15.33	71.32 ± 11.89	62.07 ± 15.64	54.45 ± 13.25	45.55	0.167	<0.001	C1 > C2 > C3

C1, Surface Acting–Suppression Type; C2, Deep Acting Type; C3, Natural Engagement Type.

*Post hoc* analysis revealed that nurses in the Surface Acting–Suppression Type group (C1) had the highest levels of psychological resilience, followed by those in the Deep Acting Type group (C2), with the lowest scores observed in the Natural Engagement Type group (C3), indicating a trend of C1 > C2 > C3.

To further quantify the magnitude of differences in psychological resilience across the three emotional labor profiles, Cohen’s d effect sizes were calculated for each pairwise comparison. The results are presented in Table 4. Effect sizes ranged from small to large, with the largest differences observed between the Surface Acting–Suppression Type (C1) and Natural Expression–Adaptive Type (C3), particularly for the tenacity and optimism dimensions.

**Table 4:** Cohen’s *d* effect sizes for psychological resilience dimensions across latent emotional labor profiles among nurses.

Type	*d* (C1 vs. C2)	*d* (C1 vs. C3)	*d* (C2 vs. C3)
Tenacity	0.59	1.13	0.43
Strength	0.57	1.12	0.44
Optimism	0.26	0.56	0.29
Psychological resilience	0.65	1.34	0.53

C1, Surface Acting–Suppression Type; C2 = Deep Acting Type; C3 = Natural Engagement Type.

### 3.5 Multivariate analysis of latent emotional labor profiles among nurses

Taking the Surface Acting–Suppression Type (C1) as the reference group, a multinomial logistic regression analysis was conducted. Variables that showed statistically significant differences in the univariate analysis of variance were included as independent variables.

The results indicated that gender, age, employment type, number of night shifts, salary satisfaction, and psychological resilience were significant predictors of emotional labor profile membership (*p* < 0.05). Compared to the C1 group, nurses who were female, younger, formally employed, had fewer night shifts, higher salary satisfaction, and higher levels of psychological resilience were more likely to be categorized as Deep Acting Type (C2) or Natural Engagement Type (C3).

Psychological resilience significantly differentiated between C1 and C2, C1 and C3, as well as C2 and C3, indicating its strong predictive role in profile classification.

The coding of independent variables is shown in Table 5, and the results of the regression analysis are presented in Table 6.

**Table 5:** Coding scheme for independent variables.

Variables	Mode of assignment
Gender	0 = Male, 1 = Female
Age	1 = ≤25 years, 2 = 26–35 years, 3 = 36–45 years, 4= > 45 years
Marital status	1 = Married, 2 = Unmarried, 3 = Others
Employment type	0 = Contractual, 1 = Permanent
Monthly night shifts	1 = ≤4、2 = 5–8、3 = ≥8
Salary satisfaction	1 = Dissatisfaction, 2 = General, 3 = Satisfied
Psychological resilience	Continuous variables

**Table 6:** Multinomial logistic regression analysis of predictors of latent emotional labor profiles among nurses (*n* = 458).

Type	Reference	*β*	*SE*	Wald *χ^2^*	*P*	*OR*	95%*CI*
C2 *vs.* C1							
Constant		1.573	0.850	3.431	0.064	–	–
Gender	Female	−1.200	0.344	12.202	0.000	0.301	0.154~0.591
Age	>45 years						
26-35 years		1.310	0.379	11.960	0.001	3.706	1.764~7.787
36-45 years		1.007	0.329	9.384	0.002	2.737	1.437~5.212
Married	Unmarried/Others	1.110	0.408	7.389	0.007	3.035	1.363~6.758
Contractual	Permanent	0.587	0.266	4.872	0.027	1.798	1.068~3.028
Psychological resilience	–	−0.047	0.009	25.429	<0.001	0.954	0.936~0.971
C3 *vs.* C1							
Constant		4.002	0.961	17.343	<0.001	–	–
Gender	Female	−1.056	0.443	5.682	0.017	0.348	0.146~0.829
Number of night shifts ≤4	≥8	−0.998	0.438	5.195	0.023	0.369	0.156–0.869
Salary satisfaction	Satisfied						
Dissatisfaction		1.268	0.512	6.121	0.013	3.553	1.301~9.701
General		0.861	0.331	6.751	0.009	2.365	1.235~4.527
Psychological resilience		−0.079	0.011	49.707	<0.001	0.924	0.904~0.944
C3 *vs.* C2							
Constant		2.426	0.817	8.813	0.003	–	–
Age	>45 years						
26–35 years		−0.032	0.009	12.401	0.000	0.969	0.951~0.986
36–45 years		−1.118	0.376	8.817	0.003	0.327	0.156~0.684
Number of night shifts ≤4	≥8	−1.108	0.366	9.186	0.002	0.330	0.161~0.676
Salary satisfaction	Satisfied						
Dissatisfaction		1.005	0.391	6.610	0.010	2.732	1.270~5.879
General		0.865	0.297	8.512	0.004	2.376	1.328~4.248
Psychological resilience		−0.032	0.009	12.401	0.000	0.969	0.951~0.986

C1, Surface Acting–Suppression Type; C2, Deep Acting Type; C3, Natural Engagement Type.

## 4 Discussion

### 4.1 Current status of nurses’ emotional labor

The present study found that clinical nurses generally experience a relatively high emotional labor burden, with surface acting being used most frequently. Approximately 30.3% of nurses were categorized as the Surface Acting–Suppression Type, indicating that a considerable proportion relied on emotional suppression and masking to meet professional service standards. While this strategy may help maintain a professional image in the short term, its long-term use can result in emotional exhaustion, role conflict, and job burnout, which is consistent with previous research (Céleste and Brotheridge, 2002; Hülsheger and Schewe, 2011).

It is noteworthy that only 24.2% of nurses were classified as the Natural Engagement Type, the lowest proportion among the three profiles. Natural expression is regarded as the most adaptive and psychologically healthy form of emotional labor (Gabriel et al., 2015); however, it often requires a supportive organizational climate and a manageable workload to emerge. Under conditions of staffing shortages and managerial pressure, it becomes difficult for nurses to express their emotions authentically and positively. This finding highlights that strengthening organizational support and fostering a more humanistic work environment remain essential tasks (Kinman and Leggetter, 2016).

### 4.2 Heterogeneity of nurses’ emotional labor profiles

This study identified three latent profiles of emotional labor among nurses using Latent Profile Analysis (LPA): Surface Acting–Suppression Type (C1), Deep Acting Type (C2), and Natural Engagement Type (C3), accounting for 30.3, 45.4, and 24.2% of the sample, respectively. These findings demonstrate marked heterogeneity in nurses’ emotional labor strategies, suggesting variation in psychological regulation styles, adaptive capacities, and occupational health statuses among nurses.

(1) Surface Acting–Suppression Type (C1)

Nurses in this group exhibited the highest scores on surface acting and the lowest on deep acting and natural expression, indicating a strong tendency to suppress genuine emotions to meet professional demands. While such strategies may support short-term role performance, they often lead to emotional exhaustion, role conflict, and burnout over time (Jin et al., 2025; Zhou et al., 2025). Interestingly, despite these risks, nurses in this group reported the highest psychological resilience. This seemingly contradictory result may reflect a form of adaptive resilience developed under sustained emotional pressure in high-intensity clinical environments. According to Conservation of Resources (COR) theory, individuals who are frequently exposed to emotionally draining work may activate compensatory psychological resources, such as resilience, to sustain professional functioning (Yikilmaz et al., 2024; Saei et al., 2024). Thus, C1 nurses may be using resilience as a buffer, but the long-term sustainability of this strategy remains uncertain. Targeted interventions are still needed to reduce surface acting demands and promote more authentic emotional regulation strategies.

(2) Deep Acting Type (C2)

Nurses in this category scored moderately to highly across all strategies, with deep acting being the most prominent. This suggests that they are able to actively regulate their internal emotional experiences to achieve consistency between inner feelings and outward expression. Compared with surface acting, deep acting is more closely aligned with emotional congruence, thereby reducing cognitive dissonance and emotional conflict, and is regarded as a more adaptive strategy of emotional labor (Pinkawa and Dörfel, 2024). Empirical evidence has shown that nurses who adopt deep acting tend to exhibit higher job satisfaction, self-efficacy, and organizational commitment (Feng et al., 2024; Xu and Fan, 2023; Değirmenci Öz et al., 2023). Although this strategy requires considerable emotional effort, it often elicits positive feedback and affective recognition from patients, colleagues, and the organization, which facilitates resource restoration and enhances emotional resilience. Consequently, deep acting has been associated with reduced work stress and lower levels of emotional exhaustion in previous research (Kinman and Leggetter, 2016; Zhang et al., 2024). This group reflects strong professional adaptability and represents a relatively healthy mode of emotional labor worthy of encouragement and reinforcement.

(3) Natural Engagement Type (C3)

The C3 group obtained the highest scores in natural expression, suggesting that these nurses were able to express their genuine emotions authentically, displaying high emotional congruence and authenticity in clinical practice. This type is considered the most sustainable and psychologically beneficial approach to emotional labor. According to Grandey’s theory of authentic emotional labor, natural emotional expression not only strengthens nurses’ sense of work meaning and self-identity but also alleviates emotional exhaustion, reduces job burnout, and improves both psychological well-being and job satisfaction (Céleste and Brotheridge, 2002; Grandey et al., 2012). Although Natural Engagement nurses accounted for the smallest proportion of the sample (24.2%), their strong emotional regulation capacity, stable psychological resources, and high adaptability highlight their resilience and professional consistency (Değirmenci Öz and Baykal, 2022). In practice, these nurses may shoulder greater responsibilities in emotional support and communication, and their strengths in emotional labor should be systematically recognized and cultivated at the organizational level.

In addition, some nurses displayed relatively high levels of deep acting combined with moderately elevated surface acting, reflecting a “mixed-strategy use” pattern. This suggests that nurses adaptively switch between strategies in response to complex clinical situations. While such flexibility may demonstrate strong situational adaptability (Gabriel et al., 2015), frequent strategy shifting may also deplete emotional resources, leading to “strategy fatigue” and cumulative psychological strain (Hülsheger and Schewe, 2011; Lee, 2023). Over time, this may increase the risk of emotional exhaustion and regulatory failure (Sloan, 2012). Nursing managers should actively identify such cases, provide timely emotional support, and implement preventive interventions to mitigate potential mental health problems.

### 4.3 Differences in psychological resilience across emotional labor profiles

The findings of this study revealed significant differences in psychological resilience among nurses across the three emotional labor profiles. Nurses in the Surface Acting–Suppression Type exhibited the highest resilience scores, followed by those in the Deep Acting Type, while the Natural Engagement Type scored the lowest (C1 > C2 > C3). This result suggests that psychological resilience may be differentially distributed across emotional labor strategies and coping patterns, indicating its potential relevance in distinguishing these profiles (Yu et al., 2025).

From the perspective of the Conservation of Resources (COR) theory, although nurses in the Surface Acting–Suppression Type primarily rely on surface acting in their emotional labor, their relatively high level of psychological resilience is more likely to represent a pre-existing or compensatory defensive mechanism rather than an outcome of surface acting (Li et al., 2025). When confronted with continuous emotional demands, these individuals may mobilize and invest psychological resources such as resilience to buffer against emotional exhaustion and maintain psychological functioning in challenging work environments (Feng et al., 2024; Fu et al., 2018).

In contrast, nurses classified in the Deep Acting Type demonstrated moderate levels of resilience. By actively regulating their emotions to achieve consistency between inner feelings and outward expressions, they often gain positive feedback and professional identity, which helps sustain favorable psychological adjustment (Sloan, 2012). Nurses in the Natural Engagement Type had the lowest levels of resilience. Although they display authenticity and congruence in emotional expression, their psychological resources may be more vulnerable to external organizational and environmental pressures in high-intensity clinical settings. As a result, they may have insufficient resource recovery and exhibit relatively weaker resilience when facing occupational stressors (Salehi et al., 2025; Yu et al., 2019).

Furthermore, the results of the multinomial logistic regression analysis confirmed that psychological resilience served as an independent predictor of emotional labor type. This finding suggests that resilience may be an important psychological factor associated with different emotional labor profiles and could be relevant in understanding how nurses cope with occupational stress and maintain mental health (Yu et al., 2025; Yu et al., 2019).

Therefore, nursing administrators should prioritize psychological resilience as a central target in emotional labor support strategies. By integrating organizational support, emotional regulation training, and psychological adjustment mechanisms, a multidimensional support system can be established to enhance nurses’ adaptability to emotional labor demands and to promote sustainable professional development.

### 4.4 Associations between emotional labor profiles and demographic variables

This study further examined the associations between nurses’ emotional labor profiles and their demographic characteristics. The results showed significant differences across gender, age, marital status, employment type, monthly night shifts, and salary satisfaction, suggesting that both individual traits and external contextual factors play an important role in shaping emotional labor strategies.

(1) Gender

Female nurses were more likely to be categorized as Deep Acting Type or Natural Engagement Type, whereas male nurses were more often classified as Surface Acting–Suppression Type. This difference may be closely related to gender role expectations and the process of emotional socialization. Women are generally encouraged from childhood to develop emotional recognition, empathy, and expressive abilities, which predispose them to actively regulate emotions in order to meet the implicit demands of nursing for “emotional gentleness” and humanistic care (Yikilmaz et al., 2024). In contrast, men, constrained by traditional gender norms emphasizing “rationality” and “restraint,” may be more inclined to adopt surface acting strategies to maintain a professional image while suppressing authentic emotions. Consequently, they are more likely to rely on surface acting as a coping style under occupational stress (Zhang et al., 2024; Yalçın et al., 2025; Diao et al., 2024).

(2) Age

Nurses aged 26–35 and 36–45 were more likely to be classified as Deep Acting Type, while those over 45 years were more often grouped into the Surface Acting–Suppression Type. This suggests significant age-related differences in emotional labor strategies, reflecting changes in psychological resources, professional perceptions, and coping mechanisms. Younger and middle-aged nurses are often in career growth stages, with stronger resilience and adaptability, which motivates them to engage in deep acting (Yu et al., 2025). By contrast, older nurses may experience resource depletion, fatigue, or burnout after prolonged exposure to high work stress, which weakens their ability to regulate emotions and leads them to rely on surface acting as a more “energy-saving” coping strategy (Zhang et al., 2024; Pace et al., 2023). In addition, older nurses frequently shoulder heavier family responsibilities and organizational management duties, further reducing the resources and space available for emotional recovery (Feng et al., 2024).

(3) Marital Status

Married nurses were more often categorized as Deep Acting Type, whereas divorced or widowed nurses were more likely to fall into the Surface Acting–Suppression Type. This finding highlights the importance of marital status as an indicator of social support in the selection of emotional labor strategies. Married nurses generally benefit from more stable family support systems, which provide psychological comfort and external resources to buffer work-related stress and enhance emotion regulation capacity, thereby facilitating the adoption of adaptive strategies such as deep acting (Ebrahimi et al., 2025; Temel et al., 2020). Conversely, divorced or widowed nurses may face greater life stress and emotional isolation, lacking effective compensatory resources, which may push them toward surface acting as a more consuming coping strategy that maintains professional image at the cost of increased psychological burden (Xu and Fan, 2023; Li et al., 2019).

(4) Employment Type

Permanent nurses were more likely to be categorized as Deep Acting Type, while contractual nurses were more often classified as Surface Acting–Suppression Type. This suggests that organizational security may influence nurses’ emotional labor strategies. Job stability, salary, and professional identity associated with permanent employment can be regarded as external resources that enhance nurses’ willingness and capacity to invest in deep acting (Ren et al., 2024; Gao et al., 2024). By contrast, contractual nurses face greater job insecurity, limited promotion opportunities, and fewer benefits, along with heavier psychological stress and resource shortages. Consequently, they are more prone to rely on surface acting as a low-cost coping strategy, which increases the risk of emotional exhaustion (Zhang et al., 2022; Liu et al., 2025).

(5) Monthly Night Shifts

Nurses working eight or more night shifts per month were more likely to be categorized as Surface Acting–Suppression Type, whereas those with fewer night shifts (≤4 per month) were more commonly found in the Deep Acting and Natural Engagement Types. This indicates that night shift workload exerts a significant influence on emotional labor strategies. Frequent night shifts are often accompanied by circadian rhythm disruption, poor sleep quality, and cumulative physical fatigue, all of which diminish emotional recovery capacity and increase stress and exhaustion risk (Xie et al., 2025). In such circumstances, nurses may find it difficult to engage in the resource-intensive process of deep acting, instead relying on surface acting as a less demanding strategy (Cheng et al., 2023). Furthermore, high-frequency night shifts limit opportunities for restorative activities such as socializing, exercise, and rest, creating a vicious cycle of “high workload and low regulation” (Yu et al., 2025; Lim and Kim, 2025). In contrast, nurses with fewer night shifts enjoy greater opportunities for recovery and access to support resources, making them more capable of adopting healthier emotional labor strategies (Li et al., 2024).

(6) Salary Satisfaction

Salary satisfaction was also significantly associated with emotional labor profiles. Nurses who reported higher salary satisfaction were more likely to be classified as Deep Acting Type or Natural Engagement Type, while those dissatisfied with their salaries were more often grouped into the Surface Acting–Suppression Type. According to the COR theory, salary functions as an important external resource that shapes perceptions of job reward and regulates emotional labor strategies (Meng et al., 2023). Higher salary satisfaction enhances nurses’ psychological security and sense of belonging, motivating them to invest more resources in achieving emotional congruence. In contrast, salary dissatisfaction may be associated with perceptions of resource loss, which in turn may relate to increased negative emotions and a greater likelihood of relying on surface acting as a lower-cost coping strategy (Kuo et al., 2025; Wen et al., 2022).

## 5 Conclusions and management implications

### 5.1 Conclusion

Based on Latent Profile Analysis, this study revealed significant heterogeneity in nurses’ emotional labor, which could be classified into three distinct profiles: Surface Acting–Suppression Type, Deep Acting Type, and Natural Engagement Type. Among these, the highest level of psychological resilience was observed in the Surface Acting–Suppression Type, followed by the Deep Acting Type, with the lowest level in the Natural Engagement Type (C1 > C2 > C3). Gender, age, employment type, monthly night shifts, salary satisfaction, and psychological resilience were identified as independent predictors of emotional labor profiles.

These findings indicate that nurses are not a homogeneous group in terms of emotional labor. Differences in psychological characteristics and resource reserves across profiles are significant. Psychological resilience plays a pivotal role in distinguishing emotional labor types and represents an important target for future interventions.

### 5.2 Management implications

(1) Stratified interventions based on profile identification

A screening and profiling mechanism for emotional labor should be established and incorporated into annual psychological health assessments. Nurses can be categorized into Surface Acting–Suppression, Deep Acting, and Natural Engagement profiles. For those in the Surface Acting–Suppression Type, special attention should be paid to workload and psychological status, with regular psychological counseling and risk monitoring implemented.

(2) Strengthening resilience training and support

For nurses classified as Deep Acting or Natural Engagement Types, individualized training programs such as emotional regulation workshops, mindfulness-based stress reduction, and empathic communication sessions should be designed to enhance emotional adjustment capacity. Authentic emotional expression should be encouraged to reduce overreliance on surface acting.

(3) Optimizing organizational environment and external resources

Scheduling systems should be improved to reasonably limit night shift frequency. Fairness in salary distribution and professional recognition should be enhanced. A sustainable organizational support system should be established, including psychological care platforms, peer support networks, and career development pathways, to help nurses restore and accumulate psychological resources.

(4) Promoting institutionalized management

Emotional labor management should be integrated into nursing quality control and occupational health management. A closed-loop mechanism of “screening–intervention–evaluation–feedback” should be established to achieve coordinated improvement of nurses’ mental health and organizational development.

## 6 Limitations and future research directions

### 6.1 Limitations

This study has several limitations. First, its cross-sectional design does not allow for causal inferences regarding the relationship between emotional labor profiles and psychological resilience. Although causality cannot be determined, cross-sectional designs still hold significant value in identifying synchronous associations between psychological variables. This is particularly relevant to the present study, which employed latent profile analysis (LPA) to explore the heterogeneity of emotional labor among nurses. LPA is well suited for uncovering latent subgroups in cross-sectional data and generating hypotheses for future longitudinal research. Future studies are needed to validate the explanatory pathways or associations identified in this study.

Second, the sample was drawn exclusively from eight tertiary hospitals in Sichuan Province, which may limit regional and institutional representativeness. Due to the non-probabilistic sampling strategy, the findings cannot be generalized beyond the study sample. Future research should employ probabilistic sampling across multiple regions and healthcare levels to improve generalizability.

Third, although validated self-report instruments were used, the absence of confirmatory factor analysis (CFA) limits the ability to formally verify the structural validity of the scales within this sample. In addition, despite the use of Harman’s single-factor test suggesting that common method variance (CMV) was not a major concern (first factor = 28.44%), reliance on self-reports may still introduce bias. Future studies should incorporate CFA and consider multi-source or objective measures (e.g., peer ratings, physiological data) to enhance measurement validity and reduce method-related bias.

Finally, this study did not include potential mediating or moderating variables (e.g., organizational support, job burnout, job satisfaction), limiting deeper exploration of emotional labor mechanisms. Additionally, some context-related variables (e.g., workload intensity, department type), though theoretically relevant, were either unmeasured or excluded due to nonsignificant effects. Future research should incorporate these factors to provide a more comprehensive understanding of emotional labor among nurses.

### 6.2 Future research directions

Future research can be advanced in the following ways:

(1) Adopt longitudinal or mixed-methods designs to explore the dynamic changes and causal relationships between emotional labor profiles and psychological resilience over time;
(2) Expand the sampling framework to include nurses from multiple regions, levels of healthcare institutions, and specialties, thereby improving the generalizability of findings;
(3) Introduce objective indicators (e.g., physiological stress responses, workload monitoring) in combination with multidimensional assessment tools to increase the precision of measurements of emotional labor and resilience, and to better capture context-related variables such as perceived workload intensity or staffing levels;
(4) Further examine the mediating or moderating roles of organizational support, leadership style, interpersonal relationship quality, and work environment characteristics (e.g., department type or shift patterns) to construct a more comprehensive theoretical model;
(5) Integrate intervention studies to evaluate the effectiveness of profile-based stratified interventions in enhancing resilience and reducing the negative effects of emotional labor.

## Data Availability

The raw data supporting the conclusions of this article will be made available by the authors, without undue reservation.
